# Zeroing in on AIDS and global health Post-2015

**DOI:** 10.1186/1744-8603-8-42

**Published:** 2012-11-30

**Authors:** Kent Buse, Ruth Blackshaw, Marie-Goretti Harakeye Ndayisaba

**Affiliations:** 1 Political Affairs and Strategy, UNAIDS Secretariat, Geneva, Switzerland; 2 HIV/AIDS, Tuberculosis and Malaria Division, Department of Social Affairs, African Union Commission, Addis Ababa, Ethiopia

**Keywords:** HIV, AIDS, Post-2015, Shared responsibility Global solidarity, Health justice, Human rights

## Abstract

December 1st marks World AIDS Day with the theme ‘Getting to zero’. Three years ago, UNAIDS articulated what was then considered to be an ambitious vision, the aspiration for zero new HIV infections and zero-AIDS related deaths underpinned by zero discrimination. As we imagine the Post-2015 development agenda, we can and should reconceptualise this vision as a set of concrete goals.

This Viewpoint argues that today’s rapidly changing world, including its shifting geo-political and economic landscape, requires policy responses that are context-sensitive. We highlight the Shared Responsibility-Global Solidarity agenda, as pioneered by the African Union in its recent Roadmap on AIDS, tuberculosis, and malaria, to illustrate ways in which global health can be re-thought to tackle twenty-first century challenges. In light of the emerging debate on what a Post-2015 development agenda and accountability framework should look like, we argue that the AIDS response offers lessons as a pathfinder which can pave the way for global health responses in which the most marginalised are at the centre of the debate, human rights are protected under the rule of law, strong accountability is in place for results for people, and community and participatory processes are the norm. These hard-learned and -won principles of the AIDS response are critical if we are to realize a world in which there is zero inequality and health justice for all.

## Main text

December 1st marks World AIDS Day with the theme ‘Getting to zero’. Three short years ago, UNAIDS articulated what was then considered to be an ambitious vision – the aspiration for zero new HIV infections, zero discrimination and zero-AIDS related deaths. As we imagine the Post-2015 development agenda, we now can and should reconceptualise this vision as a set of concrete goals.

In 2011, for the first time in the history of the AIDS response, the number of people receiving antiretroviral therapy in low- and middle-income countries (just over 8 million) outweighed those needing treatment, but without access [1]. With the number of people newly acquiring HIV continuing to decline, alongside progress in the scale-up of treatment coverage and evidence of the effectiveness of treatment for prevention, achieving the ‘three zeroes’ in the coming years is possible. Indeed, at the 2012 XIX International AIDS Conference in Washington, a number of renowned experts argued that the science exists to make an AIDS-free generation a reality [2]. Given that the technical solutions are available, political will, increased investments, smart spending and scaling up proven, context-sensitive approaches are what is now needed [3].

Yet the world is changing. Many traditional providers of Overseas Development Assistance are reducing commitments in the context of the Eurocrisis and its ensuing wake of financial turmoil. In the case of the AIDS response, international assistance from donor governments and philanthropies has remained stable over the past three years at around $8.2 billion [4]. This shift comes at a time when emerging economies, such as Brazil, Russia, India, China and South Africa (the “BRICS”), are rapidly increasing domestic investments in AIDS while leveraging their increasing economic and political clout to forge new approaches to development cooperation. Africa is experiencing unprecedented economic growth and many countries are investing considerably in industry and social services – a change well illustrated by *The Economist’s* shift from labelling Africa ‘the hopeless continent’ [5] in 2000, to its assessment in 2011 of ‘the hopeful continent – Africa rising’ [6]. It is these changing geo-political and economic realities which must be reflected in charting a new path for AIDS and global health in the Post-MDG development agenda – one which integrates the economic, social and environmental pillars of development in a bid to move towards sustainable, people-centred, equitable and holistic solutions.

AIDS has always been a pathfinder and it can continue to blaze a trail as the international community articulates what sustainability means and how to achieve it in a Post-2015 framework. Jim Yong Kim, President of the World Bank, alluded to this potential when he encouraged the international community to “join me in harnessing the moral power and practical lessons that the AIDS movement has produced to speed progress against that other global scourge, poverty” [7]. Experience drawn from the global AIDS response can contribute to doing development differently across all three pillars of sustainable development. It can guide the journey towards development in which: people are agents of change – with the most marginalised in the driving seat of the debate; human rights are protected under the rule of law; strong accountability is in place for results for people; and community mobilisation and inclusive and participatory processes are the norm – recognising that the process is as important as the end result.

The potential of AIDS to act as a pathfinder is being seized by African leaders. As articulated in the African Union’s *Roadmap on Shared Responsibility and Global Solidarity for AIDS, Tubercolosis and malaria response in Africa*, AIDS can lead to system-wide solutions for Tubercolosis, malaria and other infectious diseases. More specifically, the AIDS response can pave the way for Africa to enhance its industrial platform through developing its pharmaceutical sector, while emphasising with renewed vigour the importance of inclusive processes and heightened accountability for effective health governance. The Roadmap outlines three action ‘pillars’ – diversified financing, access to medicines and enhanced governance – through which Africa together with its development partners, can deliver sustainable health across the Continent [8].

The Roadmap also pioneers the concept of shared responsibility and global solidarity as an innovative paradigm for development. Rather than a discrete agenda, shared responsibility-global solidarity provides a new way of thinking about global health now and in the years after 2015. Its following elements are particularly instructive in this respect:

Shared responsibility is not just about diversifying financing for development. In Africa, for example, it is also about developing the Continent’s pharmaceutical sector to ensure pharmaceutical security and building the knowledge economy. In this context, the African Union’s Pharmaceutical Manufacturing Plan for Africa (PMPA) should be supported and its effective implementation facilitated.

Shared responsibility entails obligations across both nations and sectors. It involves increased cooperation and the integration of efforts between the public and private sectors and civil society.

Global solidarity means working together to confront global challenges. In the case of AIDS, increased country ownership depends on the sustained commitment of global partners while the transition to greater ownership unfolds.

Neither shared responsibility nor global solidarity can be achieved in isolation. The two must come together to reinforce one another.

The power-imbalance embedded in development as charity is increasingly considered morally unacceptable. Shared responsibility-global solidarity moves away from traditional donor-recipient relationships towards novel forms of development cooperation and governance as exemplified by South-South and triangular cooperation.

Increasing interdependence and complexity mark the canvas upon which global health in a Post-2015 environment will be painted – requiring brushstrokes which are at once bold yet nuanced and flexible. Shared responsibility and global solidarity provides a context-sensitive approach to ensure sustainable and equitable results in the rapidly changing development landscape.

Achieving the ‘three zeroes’ is possible and shared responsibility-global solidarity can provide a means to get there. It can also provide new ways to re-think global health and to bring the lessons of the AIDS response behind a bid to achieve zero inequality and a world of health justice for all.

## Competing interests

The authors declare that they have no competing interests.

## Authors’ contributions

KB conceived the article and contributed to the writing; RB contributed to the writing; MHN contributed to the writing. All authors read and approved the final manuscript.
